# Commentary: Antidepressive effects of targeting ELK-1 signal transduction

**DOI:** 10.3389/fnmol.2018.00384

**Published:** 2018-10-12

**Authors:** Chang-Hoon Cho

**Affiliations:** College of Health Science, Korea University, Seoul, South Korea

**Keywords:** Elk-1, depression, chronic stress, dentate gyrus, adult neurogenesis

The prevalence and social cost of clinical depression are rapidly increasing, and the field continues to attract considerable research interest (Hayley et al., 2005; WHO executive board report, 2011).

Recently, Dr. Tzavara's group at Sorbonne University in France reported that ELK-1, a transcription factor (TF) and a major downstream target of the ERK signaling pathway, is upregulated both in depressive suicides and in animal models of depression induced by unpredictable chronic mild stress (UCMS) and social defeat (Apazoglou et al., 2018). They also found that patients who responded to antidepressant treatment had significantly lower blood levels of ELK-1 than nonresponders. Furthermore, the authors showed that virally overexpressing ELK-1 in the dentate gyrus (DG) of mice induced depression-like behaviors. Injection of blood–brain barrier-permeable TAT-DEF-ELK-1 (TDE) peptide, which disrupts ERK-mediated ELK-1 phosphorylation (activation) in the DG, improved behavioral signs in the depression models and in mice overexpressing ELK-1, without affecting basal levels of locomotion and memory (Lavaur et al., 2007; Apazoglou et al., 2018). The authors conclude that the induced expression of genes by enhanced ELK-1 activity may be critical for depressive symptoms.

The authors acknowledge that their findings contradict the current mainstream hypothesis that an overall reduction in ERK pathway activity (arising from reduced kinase activity and/or enhanced phosphatase activity) is associated with the diverse signs and symptoms of depression (see for example, Dwivedi et al., 2001; Duric et al., 2010). To explain this, Apazoglou et al. argue that cross-talk within the multiple signaling pathways involving ERK1, ELK-1, DUSP1, and the MSK1-CREB cascade is responsible for the activated network state required (and the balance in activities of signaling molecules involved) for the antidepressant effects of ELK-1 inhibition (Gutièrrez-Mecinas et al., 2011).

However, the authors did not mention other ways of regulating ELK-1 activity, which have been demonstrated in previous studies. For example, ELK-1 can be degraded by FBXO25, a ubiquitin ligase (Teixeira et al., 2013). Therefore, enhancing the interaction between ELK-1 and FBXO25, and/or the activity of FBXO25, might be alternative ways to ameliorate depressive phenotypes. It would be also worth examining the level and/or activity of FBXO25 in people with depression and in animal models to see if it is associated with enhanced expression and activity of ELK-1.

There are also other issues to be resolved around ELK-1 regulation. SIRT6, a histone deacetylase, interacts with ELK-1 to suppress ELK-1-dependent transcription (Cea et al., 2016). If ELK-1 as a TF is critical for depressive phenotypes, inhibiting SIRT6 might allow ELK-1 to work as a transcriptional activator (Besnard et al., 2011; Belzeaux et al., 2012). However, in a UCMS-induced rat model of depression, hippocampal SIRT6 expression was elevated (Mao et al., 2017). In addition, the intracellular localization of ELK-1 is not restricted to the nucleus. In neurons in particular, ELK-1 is widely expressed throughout somas, dendrites, and axonal terminals (Sgambato et al., 1998), suggesting that its activity may not be limited to that of a transcription factor. Although ELK-1's function in the cytoplasm has not been fully elucidated, there is as yet no direct evidence that its target genes are specifically involved in depression (Boros et al., 2009).

Furthermore, ELK-1 has isoforms and splice variants (Rao and Reddy, 1993; Vanhoutte et al., 2001; Kerr et al., 2010). ELK-1, which lacks domains involved in DNA binding and interaction with other TFs, has different effects to ELK-1 in DNA binding specificity and transcriptional regulation (Rao and Reddy, 1993). sELK-1 is another, shorter, isoform expressed in the brain, and increases neurite extension upon its phosphorylation, as opposed to full-length ELK-1, which does not affect neurite extension upon activation (Vanhoutte et al., 2001). The fact that sELK-1 lacks a DNA-binding domain suggests that ELK-1, not just sELK-1, is involved in processes other than gene expression. This is also supported by a report that nuclear–cytoplasmic shuttling of ELK-1 is regulated by SUMOylation (Salinas et al., 2004), which may imply that the two isoforms interact with each other, cooperatively or competitively, in response to various signals, in order to perform their cellular functions (Lavaur et al., 2007).

Besides the issues raised above, the work presented by Apazoglou et al. provides clear evidence that ELK-1 is a promising drug target for clinical depression. Interestingly, ELK-1 has been implicated in diseases comorbid with depression (Mayeux et al., 1984; Liston et al., 1987). Its expression is elevated in Alzheimer's disease (Szatmari et al., 2013), and an ELK-1/ < -synuclein interaction has been reported in Parkinson's disease (Iwata et al., 2001). Since it can be speculated that the depression experienced in these diseases results from enhanced ELK-1 activity, inhibiting ELK-1 with TDE peptide might alleviate depressive symptoms in people with these diseases.

Although the signaling pathways and molecules involved in depression have been studied extensively over the decades (Duman and Voleti, 2012), the identification of a pharmacological target with high specificity, as reported by Apazoglou et al. (2018), is particularly important. However, numerous further studies are required to determine whether ELK-1 is a meaningful target for antidepressants. First, it will be interesting to examine the level and activity of ELK-1 in newborn granule cells in animal models of depression, since ELK-1 interacts with cytoskeletal proteins to reorganize dendritic morphology and synapse maturation (Lavaur et al., 2007; Demir et al., 2009). In fact, ELK-1 was shown to contribute to mossy fiber reorganization in the hippocampus of a mouse model with traumatic brain injury (Hu et al., 2004). In addition, since the ERK pathway is involved in adult neurogenesis in the dentate gyrus (Rueda et al., 2002; Okuyama et al., 2004), it would be interesting to see if ELK-1 actively participates in the proliferation and maturation of newborn granule neurons. Second, chronic stress-induced alteration of synaptic plasticity has been reported in perforant path–dentate granule cell connections (Pavlides et al., 2002; Radahmadi et al., 2014). It will be intriguing to see what would happen to this pathway when ELK-1's activity is inhibited. Finally, it will also be interesting to examine chronic stress-induced BDNF expression when ELK-1 is inhibited (Martinowich et al., 2007).

## Author contributions

The author confirms being the sole contributor of this work and has approved it for publication.

### Conflict of interest statement

The author declares that the research was conducted in the absence of any commercial or financial relationships that could be construed as a potential conflict of interest. The reviewer HS and handling editor declared their shared affiliation at time of review.
